# Therapeutic and Preventive Potential of Plant-Derived Antioxidant Nutraceuticals

**DOI:** 10.3390/foods14101749

**Published:** 2025-05-14

**Authors:** Antia G. Pereira, Javier Echave, Ana O. S. Jorge, Rafael Nogueira-Marques, Ezgi Nur Yuksek, Paula Barciela, Ana Perez-Vazquez, Franklin Chamorro, Maria B. P. P. Oliveira, Maria Carpena, Miguel A. Prieto

**Affiliations:** 1 Universidade de Vigo, Nutrition and Food Group (NuFoG), Department of Analytical Chemistry and Food Science, Instituto de Agroecoloxía e Alimentación (IAA)—CITEXVI, 36310 Vigo, Spain; antia.gonzalez.pereira@uvigo.es (A.G.P.); javier.echave@uvigo.es (J.E.); anolijorge@gmail.com (A.O.S.J.); nogueirarafael29@gmail.com (R.N.-M.); ezginur.yuksek@uvigo.es (E.N.Y.); paula.barciela@uvigo.es (P.B.); ana.perez.vazquez@uvigo.es (A.P.-V.); franklin.noel.chamorro@uvigo.es (F.C.); 2 Investigaciones Agroalimentarias Research Group, Galicia Sur Health Research Institute (IIS Galicia Sur), SERGAS-UVIGO, 36312 Vigo, Spain; 3 Centro de Investigação de Montanha (CIMO), LA SusTEC, Instituto Politécnico de Bragança, Campus de Santa Apolónia, 5300-253 Bragança, Portugal; 4 REQUIMTE/LAQV, Department of Chemical Sciences, Faculty of Pharmacy, University of Porto, R. Jorge Viterbo Ferreira 228, 4050-313 Porto, Portugal; beatoliv@ff.up.pt

**Keywords:** phytochemicals, natural antioxidants, chemopreventive, nutraceuticals

## Abstract

Oxidative stress and its relation to the onset of several chronic diseases have been increasingly highlighted in recent years. In parallel, there has been an increasing interest in the antioxidant properties of phytochemicals. Phytochemicals are products of plant secondary metabolism, including structural polysaccharides, unsaturated fatty acids, pigments (chlorophylls, carotenoids, and anthocyanins), or phenolic compounds. Phytochemicals can be obtained from lower and higher plants, their fruits, and even from macro- or microalgae. Their diverse structural features are linked to different beneficial effects through various molecular mechanisms, contributing to disease prevention. Beyond antioxidant activity, many phytochemicals also display anti-inflammatory, antidiabetic, anti-obesity, and neuroprotective effects, which can be intertwined. Beyond these, other natural antioxidants can also be obtained from animal, fungal, and bacterial sources. Thus, a wide range of antioxidants have the potential to be used as nutraceuticals with chemopreventive effects on the onset of various diseases related to antioxidant stress. Given their enormous structural and sourcing diversity, the present work provides an updated insight into the therapeutic and preventive potential of plant-derived antioxidants and nutraceuticals.

## 1. Introduction

Phytochemicals are secondary metabolites synthesized by plants, encompassing a wide variety of chemical groups. These include phenolic compounds (PCs), fatty acids (FAs), pigments, polysaccharides (PSs), terpenoids, alkaloids, phytosterols, and saponins, among others. Their broad functional potential has positioned them as essential components in various industrial and health-related processes, including medicine, agriculture, food, textile, and cosmetic industries [1]. In particular, dietary phytochemicals have garnered increasing scientific attention in recent decades, largely due to their associated health benefits, such as cardioprotective, neuroprotective, anti-inflammatory, and gut-health-promoting effects, among others [2]. Yet, the major capacity of phytochemicals is to act as ameliorators of oxidative stress. Oxidative stress is the result of excessive levels of reactive oxygen species (ROS), which are produced by normal oxidative metabolism and exposure to the environment. The term ROS refers to molecules, such as superoxide radical (O_2_^2−^), hydroxyl radicals (OH^−^), and hydrogen peroxide (H_2_O_2_), among others. These are produced by the sequential reduction of molecular oxygen in metabolic reactions. Oxidative stress may ultimately cause damage to biomolecules and can deregulate and even disrupt physiological redox signaling, causing changes in the rate of metabolic reduction reactions, an increased rate of DNA mutations, and deregulation of cell proliferation [3]. They are also one of the major markers of cell death, which, in turn, triggers inflammation and intertwines both processes. The impact that oxidative stress has on altering normal physiological performance is known to be a factor leading to the development of inflammatory, cardiovascular, neurodegenerative, and metabolic disorders, which, in turn, may result in cancer development [4,5]. Thus, despite the presence of endogenous antioxidant systems, the intake and use of phytochemicals as significant antioxidants has been proven as a suitable alternative to improve oxidative stress status [2].

Key contributors to this activity include PCs, unsaturated fatty acids (UFAs), carotenoids, and polysaccharides, which are commonly isolated from plant-based matrices [6]. The growing recognition of these bioactivities has not only influenced dietary recommendations and favored increased consumption of fruits and vegetables, but it has also catalyzed the expansion of the nutraceutical sector. This industry now focuses on formulating products enriched with specific phytochemicals, such as purified phenolics, aimed at delivering targeted health benefits [7]. However, in recent decades, the interest in dietary phytochemicals has risen because they are thought to provide many health benefits, such as cardioprotection, neuroprotection, anti-inflammatory effects, and improved gut health, among others [2]. These effects are mainly attributed to the antioxidant properties of various groups of phytochemicals, with phenolic compounds, UFAs, and polysaccharides being chief among the key contributors [6]. Thus, diets rich in vegetables and fruits that may act as sources of phytochemicals have attracted more interest from researchers, industry, and society, with a focus on more conscious and healthy diets, as extensive research in the last decades has suggested that they are an overall healthy alternative [8,9].

To support the formulation of effective phytochemical-enriched products, it is essential to optimize extraction methodologies that maximize recovery while preserving compound integrity. Among the wide array of phytochemicals, UFAs are a particularly valuable group because of their well-documented health benefits. However, despite advances in extraction technologies, most industrial-scale studies and applications continue to rely on conventional techniques, such as solvent extraction or mechanical pressing. These approaches, while widely implemented, often present limitations in terms of selectivity, efficiency, and environmental impact [10]. UFAs, for example, can be feasibly obtained from oily crops, such as olive, sunflower, rapeseed, sesame, palm, and peanut [11], although their composition and concentration vary depending on the plant source and extraction technique. Beyond these conventional crops, several underutilized fruit seeds, including those of pepper, grape, and avocado, have also been identified as promising UFA sources [12,13]. Additionally, microalgae have gained increasing attention in recent years as sustainable and efficient producers of UFAs with high nutritional value [14].

In addition to UFAs, pigments represent another important class of phytochemicals with significant health-promoting properties and broad industrial applicability, primarily due to their antioxidant activity and their function as natural colorants [15]. Among these, carotenoids stand out as the principal group of pigments used in industrial sectors. Carotenoids can be distinguished between carotenes, which include carotene and lycopene, and xanthophylls, with some main examples being lutein, zeaxanthin, and lycopene [15]. Moreover, they can be further distinguished between provitamin A carotenoids, which include carotenes and β-cryptoxanthin, and non-provitamin A carotenoids, including mainly xanthophylls, like lycopene, lutein, and zeaxanthin [16]. Intuitively, carotenoids can be obtained from yellow, orange, and red-colored fruits and vegetables, with higher concentrations in their peels. In recent years, both seaweeds and microalgae have become feasible and cost-effective sources of carotenoids, with some major examples being fucoxanthin from brown seaweeds and astaxanthin from red microalgae [17]. In this sense, carotenoids have been highlighted as relevant phytochemicals with various potential health benefits, ranging from their nutritional value as precursors of vitamin A to health-related properties, such as antioxidant, metabolic modulation, anti-inflammatory, and neuroprotective [18].

Another important class of phytochemicals is PSs, which play a critical role in both plant biology and human health. Among them, cellulose, hemicellulose, and pectin are the major PSs present in plants, which constitute dietary fiber [19]. These structural PSs may be found more abundantly in the “harder” parts of plants, such as seeds, leaves, and peels, and are of high interest as promoters of healthy gut microbiota [20]. Conversely, storage polysaccharides (that act as stored energy), which include starches and other PSs (i.e., fructans), are more abundant in bulbs, tubers, and pulps [21].

Lastly, PCs are antioxidant phytochemicals of paramount importance that have been exhaustively studied in the last two decades and have a ubiquitous distribution in the plant kingdom. PCs are mainly synthesized in plants in response to biotic (predators, infections) or abiotic (drought, salinity) factors [3]. Their diverse structural complexity and degree of polymerization also determine their bioavailability, activities, and occurrence. Some well-known examples of PCs and sources include flavanols in grapes or tea, flavonols in citrus, and anthocyanins in berries [3,22].

Given their vast variety, sources, and beneficial effects related to health, natural antioxidants have been increasingly used as nutraceuticals. Although these diverse molecules have been generally observed to display null or usually lower toxicity than synthetic or conventional chemicals, they may also display some undesired effects. For instance, it is known that PCs establish synergies in their antioxidant effects when various diverse molecules are present; however, when a single compound is present at high concentrations, PCs may act as prooxidant molecules, a fact that has also been observed with other antioxidant molecules [2]. Moreover, as with every other nutrient and chemical, an excessive intake can lead to adverse effects on health. For example, excessive intake of β-carotene has been associated with an increase in serum triglycerides, cholesterol, and bone loss [23]. On the other hand, ROS and other reactive species that are produced as a result of physical activity are needed for endogenous responses that enable muscle growth, which antioxidant supplementation may partially hurdle [24]. Nonetheless, beyond potential hepatotoxicity if the daily consumption is consistently very high, negative side effects of these types of natural compounds are limited, and mainly attributed to other elements, especially metals [25,26].

As research continues to uncover the full extent of their health benefits, these compounds are increasingly recognized for their potential to act as antioxidants, support disease prevention, enhance immune function, and improve overall well-being. As interest in these compounds continues to grow, there is a shift towards more economically viable production methods, including chemical and microbial synthesis, which offer greater consistency and scalability [27]. Additionally, the extraction of phytochemicals from natural sources, particularly through the use of agri-food waste from fruits and vegetables, is emerging as a sustainable and cost-effective approach [28]. Although these methods may be cost-effective, chemical extraction of phytochemicals from their natural sources is becoming increasingly widespread, especially using fruit and vegetable agri-food waste as a source of phytochemicals [28]. The aim of the present work is to discuss the role of antioxidants in plants, their significance in the mitigation of oxidative damage, and their implications for plant resilience and human overall health through other beneficial effects on health, such as anti-inflammatory, antidiabetic, anti-obesity, and neuroprotection. Although this work focuses on UFAs, PCs, PSs, and carotenoids, various other natural compounds with antioxidant properties, as well as significant benefits to health and potential use as nutraceuticals, are also explored.

## 2. Chemical Classes of Antioxidant Phytochemicals: Structural Significance

### 2.1. Unsaturated Fatty Acids

**Figure 1:**
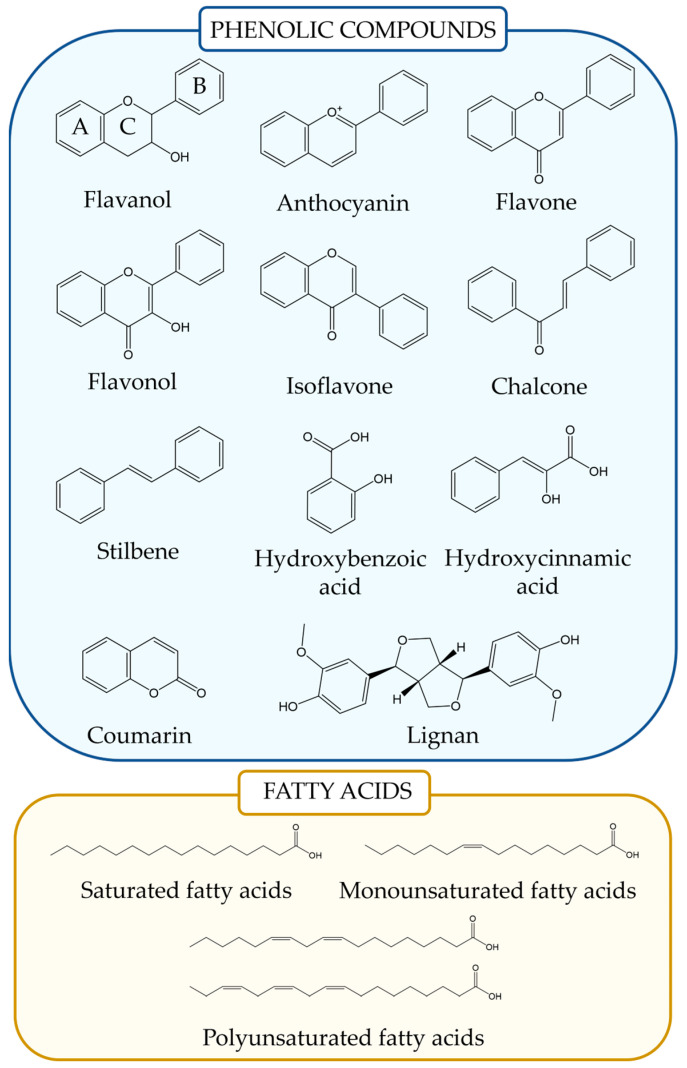
Chemical structure and examples of phenolic compounds and fatty acids.

FAs are hydrophobic aliphatic carbon chains of variable length that contain a carboxyl (-COOH) group at the end of the chain [29]. FAs are classified by the number of carbons in the chain, but they mainly depend on the presence of double bonds between the carbons (Figure 1). FAs with no double bonds present are called saturated FAs (SMFAs), while those with double bonds are called UFAs, which are further classified based on whether they have a single bond (monounsaturated FAs, MFAs) or two or more bonds (polyunsaturated FAs, PUFAs) [30]. In addition, fatty acids can also be distinguished depending on their stereochemistry, presenting *cis* or *trans* isomers, with *cis* being more prevalent in plants and *trans* more prevalent in animal fats [31]. Depending on the position of the double bonds in the chain, UFAs can be further distinguished as omega (ω). In the case of plant organisms, fatty acids may usually present a length in the range of 12 to 22 carbons, with 16 and 18 carbons being generally the most abundant [32]. Among MFAs, plant organisms generally accumulate palmitic (C16:0) and stearic (C18:0) acids. Moreover, plants are the main source of UFAs in nature and, thus, in the human diet, with some major representatives being oleic acid (C18:1) and linoleic acid (C18:2) [11].

### 2.2. Phenolic Compounds

As mentioned, PCs are produced by plants primarily in response to environmental stress factors, such as drought, extreme temperatures, UV exposure, pollution, and pathogen attacks [33], and their biosynthesis follows pathways like the pentose phosphate, shikimate, and phenylpropanoid routes [34]. Chemically, PCs are defined by the presence of one or more hydroxyl groups attached to an aromatic ring, though their complexity varies across different subclasses. These compounds encompass a broad range of molecules, including phenolic acids, flavonoids, stilbenes, lignans, and related structures, like chalcones, humulones, and alcohols [33,35]. These compounds are classified according to their structure, with more than 8000 variations currently identified. The classification is typically based on the number of phenolic rings and the nature of the linkages between them. The major groups include phenolic acids, flavonoids, stilbenes, and lignans [36].

Phenolic acids are generally the most ubiquitous and the smallest PCs in terms of molecular size and weight, and they are divided into two main categories: hydroxybenzoic acids (e.g., gallic acid) and hydroxycinnamic acids (e.g., caffeic, ferulic, coumaric acids) [36,37]. Flavonoids, another key category, have a C6–C3–C6 backbone and are further categorized into subgroups (Figure 1), such as flavonols (e.g., quercetin, kaempferol, morin, myricetin), flavones (e.g., luteolin, acacetin, chrysin, baicalein, wogonin), isoflavones (e.g., genistein), anthocyanins, flavanones (e.g., naringenin, hesperetin), flavanols, and chalcones (e.g., xanthohumol isoxanthohumol, isobavachalcone, licochalcone A) [37]. In plant cells, flavonoids are typically stored as glycosides, and their antioxidant potential is influenced by the degree of glycosylation. Aglycones, like quercetin and myricetin, are generally more potent antioxidants than their glycoside forms, but with hampered bioavailability [38].

Tannins, another class of phenolics, are mainly categorized into hydrolysable tannins (gallotannins, ellagitannins) and condensed tannins and phlorotannins (based on phloroglucinol, which is only found in brown seaweeds). These compounds are known for their protein-binding capabilities, contributing to the astringent taste of certain fruits and their antimicrobial activities [36,39].

Additionally, some PCs, like curcumin (diferuloylmethane), display a broad spectrum of biological functions, including antioxidant, anti-inflammatory, antimicrobial, antitumor, and liver-protective effects [40]. The structure of PCs is closely linked to their bioactivities, with different chemical arrangements providing specific biological functions [41,42].

Given their broad spectrum of bioactivities, PCs are increasingly being incorporated into nutraceuticals and functional foods aimed at improving human health and preventing chronic diseases [43]. Furthermore, these compounds contribute to the sensory qualities of many plant-based foods and beverages, particularly in terms of flavor and color [44]. In the context of human nutrition, PCs are known for their strong antioxidant properties and represent a significant portion of the antioxidant compounds in plants [45]. In terms of dietary applications, phenolic acids are commonly found in functional foods because of their beneficial health effects, such as reducing inflammation, preventing allergic reactions, and offering cardiovascular protection [46]. As research continues to elucidate the specific mechanisms underlying their health benefits, phenolic compounds hold promise as key ingredients in the development of novel therapeutic agents and functional food products [46,47]. Additionally, phenolic compounds have extensive applications in the food, cosmetic, and pharmaceutical industries, where they are used for their antioxidant and preservative properties, as well as their ability to improve the stability and shelf life of products [2]. They also represent a significant portion of the antioxidant content in plant-based foods [45].

### 2.3. Carotenoids

**Figure 2:**
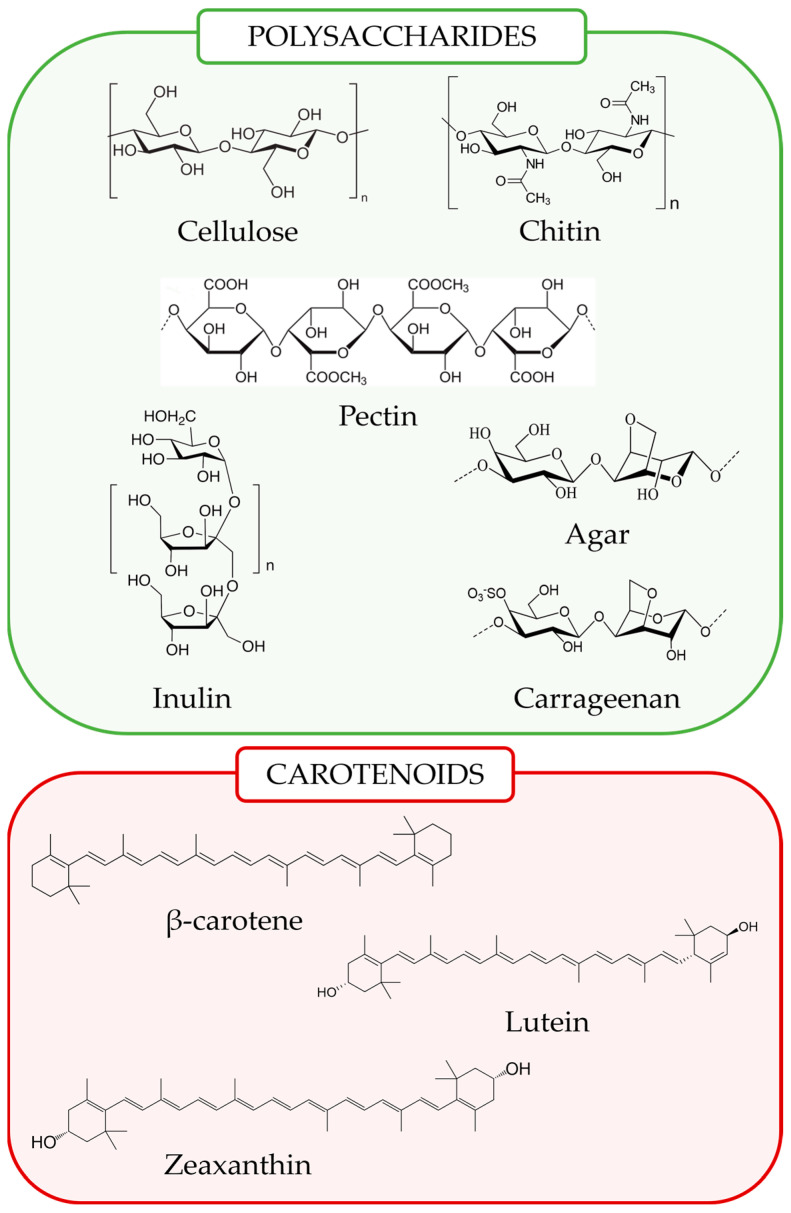
Chemical structure of some major examples of polysaccharides and carotenoids with beneficial health effects.

Given their nature and metabolic function as reducing agents, plant pigments are used as colorants and antioxidants. Carotenoids represent a class of pigments naturally produced by plants, fungi, algae, and specific bacteria [48]. These compounds are responsible for the vibrant red, yellow, and orange colors of various fruits and vegetables [49]. Structurally, carotenoids are classified as tetraterpenoids, composed of a 40-carbon polyene chain that provides their antioxidant properties (Figure 2) [50]. The conjugated double bond system present in the structure of carotenoids facilitates electron delocalization, thereby increasing their ability to neutralize free radicals and singlet oxygen [51]. Additionally, their antioxidant efficiency is modulated by the degree of unsaturation and the presence of functional groups, like hydroxyl (-OH) or keto oxo (C=O) groups [52].

Based on their structure, carotenoids can be divided into two primary categories: carotenes, which are purely hydrocarbons, and xanthophylls, which contain oxygenated groups [53]. The functional groups in xanthophylls generally improve their solubility and interaction with cellular membranes, potentially influencing their antioxidant effectiveness [54]. In fact, the presence of additional oxygen-containing functional groups often makes xanthophylls more potent antioxidants compared to carotenes [55].

Plants, algae, and fungi can produce different isomers of carotenes, with *α*-carotene and *β*-carotene being the most prevalent in plants (Table 1). Other isomeric forms include γ-carotene, δ-carotene, ε-carotene, and ζ-carotene. Both *α*-carotene and *β*-carotene serve as precursors to vitamin A [56], although *β*-carotene exhibits greater antioxidant activity compared to *α*-carotene [57]. Among xanthophylls, the key molecules include fucoxanthin, astaxanthin, lutein, zeaxanthin, and *β*-cryptoxanthin [53]. These compounds are recognized for their strong antioxidant properties, which play a role in their anticancer, anti-inflammatory, and neuroprotective effects, as well as their ability to enhance immune responses [53,58]. Owing to these attributes, carotenoids hold considerable potential for therapeutic use in managing OS-related diseases [59]. The variation in their efficacy stems from differences in their structural characteristics, such as terminal functional groups and chain length [53]. For instance, lutein and zeaxanthin, commonly found in leafy greens, are known to accumulate in the retina’s macular region, where they filter harmful blue light and serve as powerful antioxidants [58].

Additionally, several studies support the inclusion of carotenoids in functional foods and dietary supplements, linking their intake to a reduced risk of chronic diseases, including cardiovascular disorders, specific cancers, and age-related macular degeneration [49]. Their role as precursors to vitamin A further enhances their significance in promoting visual health and supporting the immune system [60]. The numerous benefits of these compounds have favored the inclusion of these molecules as ingredients in the development of nutraceuticals aimed at improving health and preventing diseases.

### 2.4. Polysaccharides

PSs are complex polymers composed of monosaccharide units connected by glycosidic bonds, which are widely distributed in plants, fungi, and algae [61]. These natural polymers have attracted significant attention due to their potential as nutraceuticals, particularly for their antioxidant, immunomodulatory, and anti-inflammatory activities [62,63]. Additionally, PSs prevent ROS-induced tissue damage through both free radical scavenging (Table 1) and modulation of the immune response [64,65]. However, under specific conditions, certain PSs may also exhibit prooxidant behavior, adding complexity to their antioxidant profile [66,67].

Overall, the antioxidant capacity of PSs is multifactorial, owing not only to their size but also to their structure and functional groups [68]. Structural features, such as monosaccharide composition, glycosidic linkages, molecular weight, and branching degree, contribute to their antioxidant activity [69]. Moreover, PSs with higher molecular weights, increased branching, and specific functional groups, such as sulfate or carboxyl groups, often demonstrate stronger antioxidant potential [70,71]. Moreover, the method of extraction plays a crucial role in determining their functional properties [72,73]. However, despite the established antioxidant potential of PSs, there is limited research on highly purified forms; thus, the possible influence of other bioactive components, which may be present in combination with PSs, must be taken into account [68].

PSs are classified based on their structure, origin, and function. Structurally, they can be divided into hetero-PSs (e.g., hyaluronic acid, chondroitin sulfate, heparin) and homo-PSs (e.g., fructans, galactan, glycogen) [74]. Based on their source, they are categorized into several groups: plant-based (e.g., pectin, cellulose, starch), microbial-based (e.g., curdlan, dextran, bacterial cellulose), fungi-derived (e.g., *β*-glucans, chitin derivatives), animal-derived (e.g., chitosan, chitin, heparin), and marine-derived (e.g., agar, alginate, fucoidans) [75]. PSs like pectin, which is rich in galacturonic acid, exhibit significant antioxidant properties. This bioactivity is influenced by their uronic acid content and degree of polymerization [76]. Moreover, sulfated PSs from marine algae are particularly effective in neutralizing free radicals and chelating metal ions, enhancing their antioxidant performance [77]. Ongoing research continues to shed light on the mechanisms by which these PSs exert their health-promoting effects, opening new avenues for their use in preventive and therapeutic nutrition [78]. Moreover, beyond their role as prebiotics, PSs are also widely utilized in industry for various functions, including as thickeners, emulsifiers, stabilizers, gelling agents, and controlled-release agents, broadening their applications [74,78].

**Table 1:** Concentration and antioxidant activity of antioxidant phytochemicals in principal sources: data from chemical assays.

Class	Compound	Main Source	Concentration	Assay	AA	Ref.
UFAs *						
ω-3	ALA	Olive, sunflower, linseed, rapeseed, fruit and vegetable seeds, other oily crops	5.5–61.5%	ROS	16.86 mM	[79,80,81,82]
	EPA	Seaweed, microalgae, fish oil	6.6–22.5%	ROS	150 µM	[83,84]
	DHA	Seaweed, microalgae, fish oil	1–6.6%	ROS	100 µM	[83,85,86]
ω-6	LA	Olive, sunflower, linseed, rapeseed, nuts, fruit and vegetable seeds, other oily crops	16.5–62.5%	ROS	39.5 mM	[79,82,87,88,89]
ω-7	PA	Olive, nuts, macadamia nuts, microalgae	0.6–50.1	–	–	[90,91,92,93,94]
ω-9	OA	Microalgae, linseed, rapeseed, nuts, fruit and vegetable seeds, other oily crops	1.4–79.6%	SOD	53.1 mM	[82,83,91,95,96,97]
**Carotenoids**						
Carotenes	*α*-carotene	Carrots, pumpkins	13.44–30.11 mg/kg fw	ROS	40.6 µmol TE/g dw	[98,99]
	*β*-carotene	Carrots, red peppers, oranges, potatoes, green vegetables	41.60–71.2 mg/kg fw	ROS	7.2 µmol TE/g dw	[98,99]
Xanthophylls	Fucoxanthin	Brown algae	0.02–18.60 mg/g dw	ROS	201 μg/mL	[53,100]
	Astaxanthin	*Haematococcus pluvialis*	3.8%	ROS	1.33 mM	[101]
	Lutein	Microalgae, algae, vegetables (i.e., kale, spinach)	0.7–5% dw	ROS	1.8–22 μg/mL	[102]
	Zeaxanthin	Red and brown seaweed, red/orange vegetables/fruits	0.49–1230 µg/g dw	ROS	2.2 μg/mL	[53,103]
	*β*-cryptoxanthin	Algae, red/orange vegetables/fruits	409–1103 µg/g dw	ROS	38.30 μg/mL	[104,105]
**Polysaccharides**						
HE	Hyaluronic acid	*Streptococcus* spp., *Tremella fuciformis*	1300 µg/mL	ROS	69.2–78.4%	[106,107]
	Chondroitin sulfate	Bacteria and cartilage	-	MCC	3.33 mg/mL	[108,109]
	Heparin	Marine organism, Asteraceae plants	-	EA	2.20 mg/mL	[110,111]
HO	Fructan	Prokaryotes, lower and higher plants	0.9–1.8 g/100 g in different wheat cultivars	EA, MCC	0.12 mg/mL	[21,112]
	Galactan	Seaweeds, seeds of some plants	-	SOD, GSH-Px	9 μM	[113]
Plant	Pectin	Cell walls of terrestrial plants	Citrus peels 30% fw, oranges 0.5–3.5% fw, carrots 1.4% fw	ROS	161.94 ppm	[114,115]
	Cellulose	Cell walls of terrestrial plants	40–50% fw	ROS	80.9 ppm	[116,117]
	Starch	Cereals, pseudocereals, legumes, root tubers	60–75% fw	ROS	97 µg/mL	[118,119]
Microbial	Curdlan	*Agrobacterium* sp., *Rhizobium* sp.	34.04 mg/g	ROS	82% DPPH, 72% ABTS	[120,121]
	Dextran	Lactic acid bacteria	580 mg/100 mL dw	ROS	97 μg/mL	[122,123]
	Cellulose	*Acetobacter* spp., *Sarcina* spp., *Agrobacterium* spp.	60.7% dw	ROS	80.9 ppm	[116,124]
Fungi	β-glucans	Fungal cell walls	31% dw	ROS, EA	161–4019 μg/mL	[125,126]
	Chitosan	Cell wall of filamentous fungi	20–45% dw	ROS	0.022 mg/mL	[127,128]
Marine	Fucoidan	Brown seaweed	20% dw	ROS	0.058 mg/mL	[129,130]
	Alginate	Brown seaweed	20–60% dw	ROS	121.4–346.3 mol/g	[131,132]
	Cellulose	Green algae	1.5–34%dw	ROS	0.15–0.39 mg/mL	[124,133,134]
**Phenolic compounds**						
Total PCs	-	*Phoenix dactylifera* var Bunarinja	34.90 mg/100g fw	ROS	0.875 μg/mL	[135]
Phenolic acids	Caffeic acid	Green coffee	6.56 μg/mL	ROS	6.31 μg/mL	[136]
	Chicoric acid	*Echinacea purpurea*	56.03 mg/g dw	ROS	15 μg/mL	[137]
	Ferulic acid	Rice bran	8.71 mg/g	ROS	9.9 μg/mL	[138,139]
Flavonoids	Myricetin	Green tea	0.40–0.79 mg/g	ROS	4.68 µg/mL	[140]
	Apigenin	*Gentiana veitchiorum*	37.50 mg/L	ROS	8.26 mg/mL	[141]
Total tannin	-	Ginger	35.08 mg/g	ROS	1 mg/mL	[142]
	-	Garlic	7.44 mg/g	ROS	3.7 mg/mL	[142]
	-	*Myristica fragrans*	14.03% *w*/*w*	ROS	89.98 μg/mL	[143]

Abbreviations: UFAs: unsaturated fatty acids; EFAs: Essential Fatty Acids; OA: oleic acid; DHA: docosahexaenoic acid; EPA: Eicosapentaenoic Acid; HEs: Heteropolysaccharides; HOs: Homopolysaccharides; PCs: phenolic compounds; dw: Dry Weight; fw: Fresh Weight; MCC: metal cation chelation; EAs: enzymatic antioxidants; ROS: reactive oxygen species; AA: antioxidant activity (IC₅₀); ns: Not Specified. * UFA concentrations are presented as % of total fatty acids.

## 3. Chemopreventive and Therapeutic Properties of Antioxidant Phytochemicals

**Figure 3:**
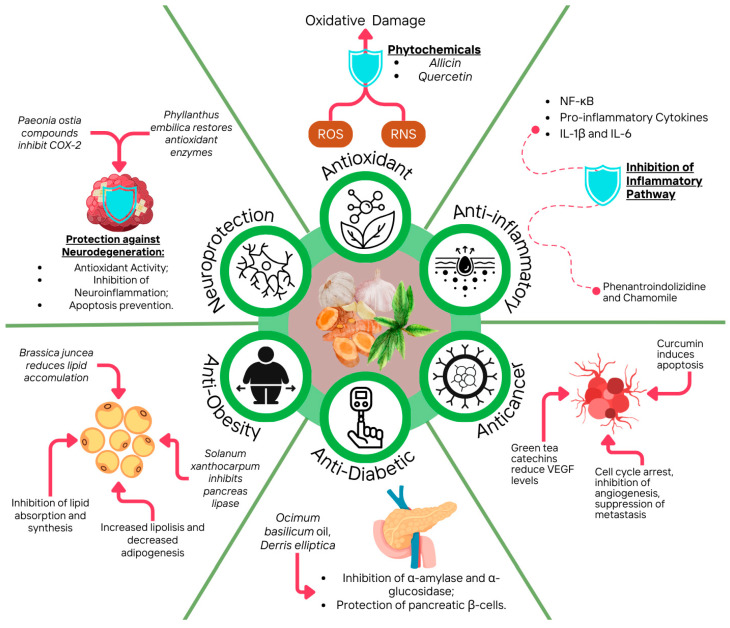
Overall view of the therapeutic properties of antioxidant phytochemicals, as illustrated with some of the examples mentioned.

Diets rich in plant-based foods are generally associated with reduced cancer risk and all-cause mortality due to their related health benefits, including antioxidant, anti-inflammatory, antidiabetic, anti-obesity, and, potentially, anticancer effects (Figure 3). This suggests that dietary phytochemicals from various plant species hold potential for cancer chemoprevention [144]. This section will explore in vivo and in vitro studies on the biological properties of phytochemicals and other antioxidants from common and uncommon sources, highlighting their effects and mechanisms described (Table 2) [145].

### 3.1. Antioxidant and Anticancer Properties

Antioxidants and phytochemicals act through two main mechanisms: chain breaking by donating electrons to free radicals and quenching ROS and other free radicals. As mentioned, there is a preeminent intertwining between oxidative stress and various diseases, including being considered a major contributor to the onset of cancer [186]. As such, various antioxidants from common and uncommon sources have been studied, considering both properties, by direct and indirect pathways (Table 2). The authors of a recent study examined the antioxidant properties of allicin, a major and fragrant sulfur organic compound from garlic, in in vitro and in vivo models of cholangiocarcinoma (CCA) [146]. Allicin was found to reduce oxidative stress and inhibit the STAT3 signaling pathway, which promotes tumor growth. The study demonstrated that allicin suppressed CCA cell proliferation, migration, and invasion while inducing apoptosis. Its dual action as an antioxidant and inhibitor of tumor-promoting signals suggests allicin could be a promising therapeutic option for CCA [146].

An in vivo study evaluated the phytochemicals in green tea (*Camellia sinensis*), including catechins and curcuma (*Curcuma longa*) curcumin, for chemopreventive effects in a hamster buccal pouch carcinoma model [144]. Green tea PCs, which mainly comprise flavan-3-ols and, mainly, epicatechin gallate, inhibited angiogenesis, while curcumin induced apoptosis through cytochrome c release and caspase activation. Both phytochemicals reduced cell proliferative and angiogenic activity, and when combined, they were more effective at inducing apoptosis and cell cycle arrest [144]. The PC-rich aqueous extract of virgin’s rose (*Fagonia cretica*) was tested for its effects on breast cancer cell lines. The results proved that the extract induced cell cycle arrest and apoptosis through both p53-dependent and -independent mechanisms. Specifically, it led to a significant increase in tumor protein p53 and FOXO3a expression [151]. On the other hand, plant-derived quercetin exhibited chemopreventive effects in HepG2 (human liver cancer) cells by reducing ROS, downregulating PI3K, PKC, and COX-2, and increasing pro-apoptotic p53 and BAX expression, highlighting its antioxidant and anticarcinogenic action [157]. One in silico and in vitro study attributed Nile tulip’s (*Markhamia lutea*) strong antioxidant properties primarily to its rich PC content, mainly including flavonoids and phenolic acids, which effectively scavenged free radicals and chelated metal ions [155].

A study on tobacco weed (*Elephantopus mollis*) highlighted 3,4-di-*O*-caffeoyl quinic acid for its significant antioxidant activity and chemopreventive properties, including apoptosis induction in NCI-H23 (human lung adenocarcinoma) cells and *β*-glucosidase inhibition [153]. Additionally, the leaf extract of *Onobrychis argyrea* has demonstrated significant antioxidant, antidiabetic, anti-Alzheimer’s disease, and anticancer in vitro properties. It induced apoptosis in human colorectal adenocarcinoma HT-29 cells by disrupting mitochondrial membranes and activating caspases, and it showed high antioxidant activity, enzyme inhibitory activities, and strong anticancer capacity [152]. 

### 3.2. Anti-Inflammatory Properties

Anti-inflammatory phytochemicals may help prevent cancer by targeting inflammation-related mechanisms. They induce cell cycle arrest by inhibiting cell cycle regulators, leading to halted cancer cell proliferation [187]. Moreover, they promote apoptosis by upregulating pro-apoptotic proteins and caspases while downregulating anti-apoptotic factors, thus facilitating cancer cell death [188]. They also promote autophagy by upregulating autophagy-related proteins and inhibit mTOR signaling, leading to the elimination of damaged cells. These combined effects decrease inflammation and suppress tumor progression [189].

It has been reported that mango (*Mangifera indica*) polyphenols (mainly gallic acid, hydroxybenzoic acid hexoside, and hydrolysable tannins, such as monogalloyl-glucoside) reduced NF-κB expression and phosphorylation in tumor necrosis factor-alpha TNF-*α*-treated MCF-12A cells, indicating anti-inflammatory effects. Phytochemicals identified in mango modulated the phosphoinositide 3-kinase/protein kinase B/mechanistic target of the rapamycin (PI3K/AKT/mTOR) signaling pathway [159]. *Commiphora leptophloeos* extract, rich in phenolic acids and flavonoids, showed anti-inflammatory effects by downregulating NF-κB/COX-2 and reducing pro-inflammatory cytokines (TNF-*α*, IL-1*β*, IL-6). This suggests its potential in the treatment of inflammatory bowel disease (IBD) [162]. Studies on the anti-inflammatory activity of *Helicteres isora* fruit extracts have been focused on its effects on pro-inflammatory mediators such as prostaglandin E2, COX-2, and TNF-*α*. Compared to celecoxib, a known COX-2 inhibitor, a hexane extract showed the strongest inhibition of PGE-2 (69.68%), followed by an ethanol extract (57.17%). All extracts were less effective than dexamethasone, a standard anti-inflammatory drug, but the hexane extract showed 51.61% inhibition of TNF-*α* [160]. The extract of *Euphorbia hirta* has been shown to have anti-inflammatory activity in vitro, which is relevant to its potential chemoprotective effects. By reducing nitric oxide (NO) production through the inhibition of inducible NO synthase (iNOS), *E. hirta* can help to lower inflammation, a known contributor to cancer advancement. Chronic inflammation can facilitate tumor growth and spread, so the extract’s ability to suppress NO and inflammatory cytokines may play a role in its anticancer properties [167]. Chamomile phytochemicals, including quercetin and the terpenoids β-amyrin and lupeol, act as NF-κB inhibitors and induce G2/M cell cycle arrest. These compounds lower pro-inflammatory cytokines IL-1*β* and IL-6 and interact with microtubules, which supports their potential as anticancer modulators of inflammatory pathways [163].

### 3.3. Antidiabetic Properties

Diabetes mellitus (DM) is a metabolic syndrome that increases the levels of glucose in the blood [173,190]. The main chronic complications of this disease are linked to the level of exposure of patients with hyperglycemia [190]. Despite the availability of chemical drugs that act as therapeutic agents, there is a need to find natural alternatives to control the complications derived from the disease and improve glycemic control and glucose intolerance [173]. In this sense, traditional medicine revealed several molecules present in plants with antidiabetic activity, as well as their mechanisms of action [190].

For instance, leaf extracts have been widely studied to determine their antidiabetic potential. An in vivo and in vitro study investigated the volatile and phenolic compounds present in basil (*Ocimun basilicum*) essential oil to determine its potential antidiabetic activity. Streptozotocin (50 mg/kg of body weight) was used as a model to induce DM in Wistar Albino rats [191]. After 28 days of feeding basil essential oil (70 mg/kg of body weight) rich in PCs, the rats showed a protective effect in pancreatic *β*-cells and a decrease in blood glucose levels. Regarding in vitro assays, the inhibitory effects on the digestive enzymes *α*-amylase and *α*-glucosidase inhibitory activity were studied, showing a strong inhibition potential. The authors linked the antidiabetic effect of basil essential oil to the phenolic and flavonoid compound content. However, the mechanism of action was not elucidated [191]. In another in vivo study, *Derris elliptica* methanolic leaf extract’s phytochemical profile and antidiabetic potential were determined [173]. Streptozotocin was used to induce DM in Sprague Dawley rats. After 14 days of feeding with the plant’s extract at two different concentrations (200 and 400 mg/kg body weight), the results showed a hypoglycemic effect and reduced cholesterol levels in a dose-dependent manner. Moreover, the highest dose increased serum insulin levels, and an antihyperglycemic effect was shown. Although the authors did not explain the mechanism of action, quercetin was the compound linked to these antidiabetic properties [173]. *Eugenia sonderiana* leaf extracts were also studied to determine their antidiabetic potential and to develop a structure–activity correlation [190]. Both in vivo and in vitro studies were assessed, which showed great *α*-amylase and *α*-glucosidase inhibitory activity and reduced glucose, pancreatic enzymes, and triglycerides, while HDL cholesterol levels were maintained within normal ranges. To explain the antidiabetic ability of *E. sonderiana* leaf extracts, the authors suggested a synergistic effect between PCs by increasing glycogen synthesis, *α*-amylase, and saponins by a complex formation with cholesterol [190].

### 3.4. Anti-Obesity Properties

Obesity contributes to the increase in the prevalence of chronic diseases, including type II DM, hypertension, dyslipidemia, cardiovascular diseases, and some types of cancer [177,192]. Common strategies to decrease the incidence of obesity are physical activity, surgery, drugs, and diet modifications [192]. In this regard, the natural compounds present in plants have been suggested as potential chemicals to be used for both the prevention and treatment of obesity [192]. Several studies have assessed the anti-obesity potential of phytochemicals. For instance, green tea has been traditionally used as a medical plant, and scientific studies have revealed its potential for obesity prevention, linked to its high content of PCs. In vitro results regarding green tea leaves suggest the anti-obesity potential of this plant by inhibiting adipocyte differentiation and proliferation [193].

In a study, C57BL/6J mice fed a high-fat diet supplemented with 0.25% Provence rose (*Rosa centifolia*) polyphenol extract for 35 days showed decreased body weight and adipose tissue, as well as reduced serum cholesterol and hepatic triglyceride levels. The treatment increased fecal triglycerides and increased lipolysis-related proteins while suppressing lipid synthesis enzymes. Ellagic acid, a key component of the Providence rose, is likely responsible for these anti-obesity effects, emphasizing its capability for treating obesity [177].

Mustard (*Brassica juncea*) attenuated lipid accumulation in 3T3-L1 adipocytes and lowered epididymal white adipose tissue mass in obese mice fed a high-fat diet [179]. Finally, acetone extract fractions from *Rumex rothschildianus*, which is rich in flavonoids and phenolics, strongly inhibited lipase activity, comparable to orlistat, and also affected *α*-amylase and *α*-glucosidase [182].

### 3.5. Neuroprotective Properties

In the past decades, there has been a rise in the incidence of neurodegenerative disorders, which has been linked to the increase in longevity that has occurred. Neurodegenerative diseases encompass a wide range of conditions that affect the central nervous system, including alterations in the neuronal structure and cellular dysfunction that lead to progressive degeneration [194]. The most prevalent are Alzheimer’s disease, Parkinson’s disease, and lateral amyotrophic sclerosis. Although there are some drugs available to ameliorate these conditions, their efficacy is limited. In the past years, natural compounds have been suggested as potential alternatives with the ability to delay disease onset, reduce disease progression, and regenerate damage via their anti-amyloid, antioxidant, and anti-inflammatory properties [195].

*Paeonia ostia* has traditionally been used as a medical and ornamental plant in China. The neuroprotective effect of the stamens of this plant has been studied by the NO inhibition assay in lipopolysaccharide-induced BV-2 cells. The (+)-3′′-methoxy-oxylactiflorin present in the plant has excellent inhibitory properties in inhibiting NO production. Moreover, molecular docking results showed that the anti-inflammatory mechanism of this molecule was a binding action with COX-2 [183]. In another study, Indian gooseberry (*Phyllantys emblica*) fruit extract, which is rich in PCs, was tested in rats with sodium valproate-induced postnatal autism. After treatment with the fruit’s extract at 100 mg/kg of body weight, the amelioration of social interactions, social affiliation, anxiety, and motor coordination was achieved. Moreover, the results showed the restoring effect of the extract in glutathione-S-transferase and glutathione reductase, which are oxidative enzymes, and a reduction in malondialdehyde (MDA) and NO levels [184]. These activities showcase its ability to induce neuroprotection by anti-inflammatory and antioxidant effects, as well.

## 4. Development of Phytochemicals as Nutraceuticals

### 4.1. Extraction, Purification, and Encapsulation

**Figure 4:**
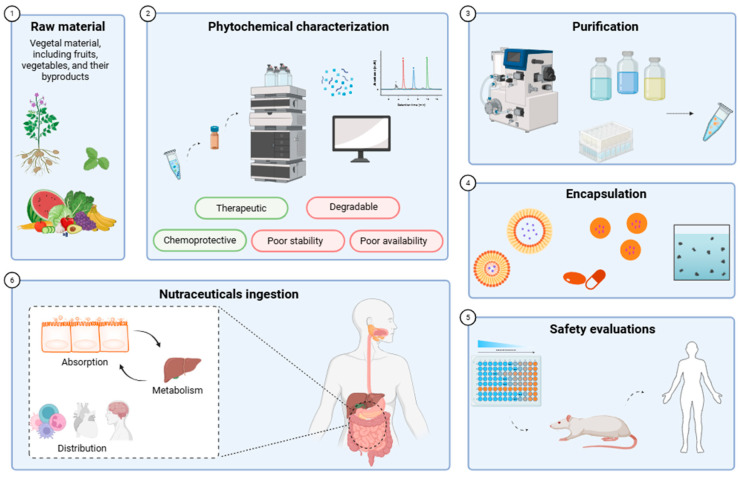
Process of development of phytochemicals as nutraceuticals.

The direct ingestion of phytochemicals within their native plant matrices presents several challenges [196]. The bioavailability of these compounds is often restricted by factors such as low concentrations, poor solubility, instability under gastrointestinal conditions, and rapid metabolic degradation and excretion [197]. These obstacles highlight the need for developing methods to concentrate, purify, and encapsulate phytochemicals, thereby enhancing their effectiveness as nutraceuticals (Figure 4) [198].

The initial phase in the utilization of phytochemicals for nutraceutical purposes involves their extraction from plant sources [199]. This extraction process is complex and must be carefully optimized to preserve the structure and bioactivity of the target compounds [200]. Traditional extraction techniques, such as solvent extraction, have been improved by emerging methods, like microwave-assisted extraction and ultrasound-assisted extraction, since they offer increased efficiency and selectivity [201]. A major challenge in phytochemical extraction and recovery is to incorporate environmental sustainability into the design and optimization of production processes, with the aim of developing more eco-friendly methodologies [202] and enhancing economic outcomes within the food industry [203]. The choice of appropriate extraction techniques is crucial, as it directly impacts the quality and characteristics of the final product [204]. The key factors influencing the efficacy of both conventional and emerging extraction techniques include the solid-to-solvent ratio, solvent concentration, particle size, and the mode of extraction (flow or batch) [205]. These parameters critically affect the efficiency of extraction and the phytochemical composition of the resultant extract [206].

Following extraction, the subsequent step involves the purification of the bioactive compounds from the crude extract. This is necessary because the initial phytochemical mixture typically contains a combination of active and inactive substances, along with impurities that could affect the safety and efficacy of the final product [207]. The degree of purification achieved is linked to the pharmacokinetic properties of the compounds, which influence their bioavailability, distribution, metabolism, and excretion [208]. Recent advancements in the purification and isolation techniques for bioactive compounds from plant sources have addressed some of the challenges posed by the complexity and diversity of phytochemicals [209,210]. These advancements focus on optimizing methods to concentrate the desired compounds, thereby enhancing bioactivity—such as antioxidant, antibacterial, or cytotoxic effects—while maintaining simplicity, specificity, and efficiency in processing [211,212]. Techniques such as high-performance liquid chromatography (HPLC), solid-phase extraction (SPE), and preparative thin-layer chromatography (TLC) are commonly used for the isolation and purification of bioactive molecules because of their practicality, cost-effectiveness, and the availability of various stationary phases [204,211]. Notably, silica, alumina, cellulose, and polyamide have proven effective in separating phytochemicals [211]. To achieve the separation of complex mixtures, the use of multiple mobile phases with varying polarities is often required [204,211].

Despite the successful purification of phytochemicals, the formulation of these compounds into nutraceuticals remains challenged by issues of stability, solubility, and bioavailability. Encapsulation technologies have emerged as vital strategies to overcome these challenges [213,214]. Additionally, encapsulation can improve the quality of food products by masking undesirable odors and flavors and extending shelf life [203]. This process involves enclosing phytochemicals within a protective matrix or carrier system, which shields them from environmental degradation, enhances their solubility in biological fluids, and facilitates controlled release in the gastrointestinal tract [215,216]. Various encapsulation methods have been developed, each offering unique advantages depending on the physicochemical properties of the phytochemical and its intended application [217]. Microencapsulation, for instance, is widely used to protect sensitive compounds and improve their stability during processing and storage [218,219]. Conversely, nanoencapsulation provides enhanced bioavailability and targeted delivery due to small particle sizes and large surface areas [220,221], which improve interactions with biological membranes and allow for more precise control over release kinetics [222,223].

In conclusion, the effective application of phytochemicals as nutraceuticals is closely linked to advances in extraction, purification, and encapsulation techniques. These processes not only enhance the pharmacological potential of phytochemicals but also ensure their stability, bioavailability, and functional integration into various nutraceutical products.

### 4.2. Considerations of Bioavailability

Phytochemicals offer a wide range of health benefits, yet their effectiveness is often compromised by challenges related to bioavailability, including issues with water dispersibility, chemical stability, and gastrointestinal absorption [224]. For phytochemicals to be effective, they must be released from their food matrices, solubilized in gastrointestinal fluids, and absorbed by enterocytes in the gastrointestinal tract. However, these compounds may precipitate, interact with other dietary molecules, or be chemically altered by digestive enzymes, metabolic pathways, or gut microbiota, which can significantly reduce their bioactive forms by the time they enter systemic circulation [225].

Therefore, the bioavailability of phytochemicals is affected by several factors, including the complexity of the food matrix, the chemical form of the compound, and individual physiological differences, such as gut microbiota composition, mucosal health, and metabolic activity. These factors contribute to considerable inter- and intra-individual variability in bioavailability, complicating the prediction of their efficacy [226]. Additionally, the impact of food processing on phytochemical stability and bioavailability is critical, as processing techniques can either enhance or diminish their bioactive potential [227]. 

To address these issues, in vitro digestion models and cellular uptake assays are employed to assess factors affecting both bioaccessibility and bioavailability. These models are essential for screening and optimizing formulations prior to more expensive in vivo studies. Despite the availability of various assessment methods, a universally accepted standard for bioaccessibility remains elusive, and the current methods must account for the diverse structural characteristics of phytochemicals [208]. For lipophilic compounds, such as carotenoids and fat-soluble vitamins, formulation techniques like encapsulation and emulsification have shown potential in enhancing bioaccessibility and protecting these compounds from degradation [208].

In silico modeling, commonly used in pharmaceutical research, offers an interesting approach as it allows for predicting phytochemical bioavailability based on physicochemical properties. However, these models are still being developed for dietary phytochemicals, indicating a need for further research to establish effective predictive tools [228]. Moreover, technological processes, such as thermal processing and biotechnological interventions, including enzymatic hydrolysis and fermentation, have demonstrated their influence in improving the bioavailability of phenolic compounds. However, while these strategies have shown promise in animal studies, further human trials are necessary to validate their effectiveness [229]. Thus, both research and innovation in processing and formulation techniques are essential for advancing nutraceuticals and optimizing the bioavailability of phytochemicals.

## 5. Current Applications of Nutraceuticals

Nutraceuticals, which combine nutritional and medicinal properties, are increasingly being incorporated into food products to promote health and prevent disease. These bioactive compounds, derived from a variety of matrices, offer a wide range of benefits, such as improving cardiovascular and immune system function and managing chronic diseases. In addition, advances in encapsulation and nanotechnology have further improved their efficacy in food applications, expanding the potential of health-promoting foods [204].

### 5.1. Ingredients in Functional Foods

**Figure 5:**
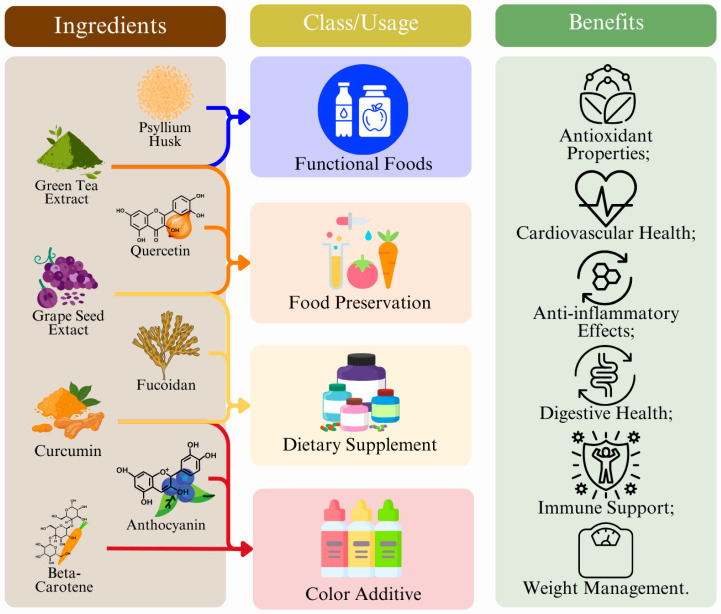
Ingredient types, uses, and benefits in food applications.

The incorporation of antioxidants into functional foods has been widely researched because of their health benefits and potential to enhance food quality and shelf life. This incorporation can be performed in several ways, including through the addition of crude extracts, fibers, color additives, and other food enrichment (Figure 5) [230].

Crude extracts are raw, unrefined substances obtained from plants, animals, and other natural sources that contain bioactive compounds. The most widely utilized are plant polyphenol extracts, such as green tea extract, grape seed extract, and rosemary extract, but also mushroom, spice, and herbal extracts (garlic extract, ginger extract, turmeric extract), vanilla extract, and, more recently, microalgae extract (Spirulina, *Chlorella* extracts) [231]. These extracts also exhibit antimicrobial activity, with green tea extract having major antimicrobial activity against major foodborne pathogens, such as *Listeria monocytogenes*, *Salmonella typhimurium*, *Escherichia coli* O157:H7, and *Campylobacter jejuni*, contributing to food safety [232,233]. Some crude extracts also contribute to flavoring, such as mushroom extracts, garlic extracts, rosemary extracts, and spice and herbal extracts, in general. These can also provide distinctive aromas and colors, enhancing their sensory attributes. For example, Spirulina extracts that are naturally greenish blue can modify the color of a food [234].

Fiber is added to foods because of its functional and health benefits. Fiber can be roughly divided into soluble and insoluble fibers. Soluble fiber includes pectin and inulin, while insoluble fiber includes cellulose, psyllium, and wheat bran. Functionally, adding fiber to foods can impact their sensory properties, such as appearance, texture, taste, and flavor [235]. Pectin can be found in fruit jams and jellies; it acts as a gelling agent, giving jams and jellies their thick, spreadable consistency. Additionally, it allows for a lower amount of sugar to be used while improving product shelf life and stability [236]. Inulin, usually extracted from chicory root, is used as a fat replacer and provides a creamy texture in low-fat or reduced-calorie foods [237]. It also serves as a prebiotic, promoting the growth of beneficial gut bacteria [238]. Cellulose and its derivatives, such as microcrystalline cellulose and bacterial cellulose, are commonly used as stabilizers and thickeners in food products, including meat products, emulsions, beverages, dairy products, and bakery items [239]. Furthermore, microcrystalline cellulose and other cellulose derivatives are used in functional and nutraceutical foods for their positive effects on gastrointestinal health and lipid metabolism [239]. Cellulose gels are also widely used in the food industry for their high-water absorption capacity and biocompatibility. They are applied in food packaging, functional foods, and food safety because of their structural flexibility and stimuli-responsive properties [240]. Other more unrefined fibers, such as psyllium husk and wheat bran, are also often used in the food industry. Unmodified wheat bran and psyllium husk are used as nutritional enhancements as they are a source of dietary fiber, minerals, vitamins, and bioactive compounds, such as phenolic acids, which contribute to improved bowel health, the prevention of diseases, like colon cancer, and cardiovascular health [241]. These fibers, when modified through processes such as mechanical milling, enzymatic hydrolysis, and thermal treatments, can be utilized as functional ingredients. For example, modified wheat bran can be incorporated into cereal foods, baked products, and fried snacks to reduce their oil content and increase their fiber content [241].

To specifically modify the color of a food, color additives can be used. The main natural color additives used in the food industry are anthocyanins, beta-carotene, betanin, and curcumin. Unlike synthetic dyes, these are usually less resistant to degradation via sunlight or heat exposure, but they do not carry risks, like causing hyperactivity in children [242]. Anthocyanins are widely used as natural food colorants because of their vibrant colors, which range from red to blue depending on pH levels [243,244]. Often, these anthocyanins are chemically modified, including acylation and glycosylation, which offers enhanced color stability, making them more suitable for industrial applications as natural colorants [245]. These also provide additional health benefits, improving carbohydrate metabolism and decreasing the risk factors of metabolic disorders [245]. Anthocyanins are used in beverages, such as fruit juices, smoothies, teas, and energy drinks, dairy products, such as flavored milk and ice creams, candies, jams and jellies, bakery products, and many others [246]. β-carotene is a pro-vitamin A carotenoid that contributes to the recommended intake of this essential nutrient in foods where it is added [60]. β-carotene is used to provide an orange hue to various food products, such as margarine and butter substitutes, fruit juices and smoothies, dairy products, such as cheese and yogurt, snacks, baked goods, and infant formula, among many others [247]. Betanin, a red-violet pigment found in beetroots, is also widely used in the food industry. Betanin exhibits high antioxidant activity, which helps to scavenge reactive oxygen species and prevent lipid oxidation in foods, thereby preserving food quality [248,249]. Betanin shows significant stability at low temperatures, which is beneficial for its use in frozen and refrigerated foods [249]. Encapsulation techniques, such as liposomal nanocarriers and microencapsulation, improve the stability and bioavailability of betanin [250,251]. Furthermore, betanin can delay the retrogradation of starches, which is beneficial for maintaining the quality and texture of starchy foods, like bread and pastries [252].

Curcumin, whether as a turmeric extract or an unprocessed form, is commonly used to impart a yellowish-orange hue to foods [253]. It has also demonstrated potential in the prevention and management of various health conditions, including cardiovascular diseases, diabetes, metabolic syndrome, arthritis, and mental disorders [254,255].

In conclusion, the incorporation of antioxidants, fibers, crude extracts, and natural color additives into functional foods has been shown to enhance not only the nutritional value but also the sensory quality, shelf life, and safety of various products. Both soluble and insoluble fibers, such as pectin, inulin, cellulose, psyllium husk, and wheat bran, play crucial roles in improving texture and stability while providing health benefits, like enhanced digestive health and disease prevention. Some examples of the mentioned nutraceuticals include Curcumin Elite™ or Mega Green Tea Extract™ from Life Extension^®^ Ltd. [256], Inülin from Töufood^®^ Ltd. [257], and Blue Spirulina from Bluetec Colorants^®^ Ltd. [258]

### 5.2. Isolated Phytochemicals as Nutraceuticals

Isolated phytochemicals are bioactive compounds derived from plants that have gained significant attention as nutraceuticals because of their potent health-promoting properties. These compounds, which include flavonoids, alkaloids, terpenoids, and polyphenols, are being increasingly isolated and studied for their potential to prevent and manage various chronic diseases, such as cancer, cardiovascular disorders, and neurodegenerative conditions (Table 3). Phytochemicals are often extracted from agricultural and food waste streams, promoting a circular economy by converting waste into value-added functional ingredients [259]. By concentrating the active ingredients, isolated phytochemicals offer more targeted therapeutic effects compared to whole plant extracts, making them valuable in the development of functional foods and dietary supplements [200].

Polyphenol extracts, which are concentrated from fruits, vegetables, herbs, and seeds, are well known for their antioxidant properties. Green tea catechins, particularly epigallocatechin-3-gallate, are effective in delaying lipid oxidation and extending the shelf life of lipid-containing foods [231,254]. These polyphenols are also incorporated into convenient products, such as chewing gum and gelatin gummies, enhancing consumer accessibility and palatability [277]. Furthermore, green tea extracts demonstrate antimicrobial properties against common foodborne pathogens, enhancing both food safety and quality [232]. Similarly, resveratrol, often sourced from grape industry waste, exhibits strong antioxidant and antibacterial activity, making it suitable for use in oil-in-water emulsions, bulk oils, and ground meat and as a potential natural preservative in food packaging and processing [278,279].

Omega-3 FAs, particularly eicosatetraenoic acid (EPA) and docosahexaenoic acid (DHA), are widely recognized for their roles in supporting brain development and cardiovascular health [280]. When sourced from marine species, such as some microalgae like *Schizochytrium* and *Nannochloropsis*, these oils are extracted through solvent methods [281]. Because of the growing consumer demand for plant-based omega-3s, microalgae supplements have become popular as they contain lower levels of heavy metals compared to traditional fish oils [282]. However, the high cost and instability of algae oils limit their use in food products, although nano-emulsification techniques have shown promise in stabilizing omega-3s for functional food applications [283]. Additionally, algae-derived omega-3s are used in livestock feed, which enriches meat, milk, and eggs with bioactive compounds that benefit human health [284], including increasing omega-3 blood indices and reducing diastolic blood pressure [285].

Fucoidans are polysaccharides extracted from brown algae that have gained recognition for their multifunctional bioactive properties. These include anticoagulant, antiviral, antitumor, antibacterial, and immunomodulatory effects [286]. Fucoidans are also valuable as gelling and thickening agents in the food industry, offering both functional and health benefits [287]. Their solubility and ease of incorporation into food formulations make them suitable for various applications, including the development of UVB-protective cosmetics and functional foods [288,289].

Agar and carrageenan, two polysaccharides derived from red algae, are widely used as gelling, thickening, and stabilizing agents in food products. Agar’s ability to form gels at low concentrations makes it ideal for candies, soups, snacks, and plant-based formulations [273,290]. Agar is also often used in plant-based and halal formulations of products that otherwise require animal gelatin [291]. Additionally, agar and its derivatives exhibit multiple bioactive functions, such as antioxidant, antiviral, antibacterial, prebiotic, antitumor, anticoagulant, and antidiabetic activities. These bioactive properties make agar a valuable ingredient in functional foods and nutraceuticals [273,290]. Carrageenan is similarly utilized as a thickening, gelling, and protein-suspending agent, enhancing texture and water retention in food products while also providing various health benefits [292]. In low-fat meat formulations, the incorporation of carrageenan promotes gel formation and water retention, thereby improving textural properties [292]. Similar to agar, carrageenan exhibits anti-inflammatory, anticoagulant, antitumor, and antithrombotic effects [293].

Quercetin, a flavonoid present in a variety of plants, is predominantly extracted from onions and apple peels because of their commercial availability [294]. In food products like bread, quercetin demonstrates antioxidant and antiglycation effects in a dose-dependent manner. Furthermore, it inhibits the formation of advanced glycation end products (AGEs), thereby enhancing the shelf life of these products [295]. Quercetin has also been shown to offer protective benefits against cardiovascular and neurodegenerative diseases, underscoring its potential as a valuable bioactive compound for the development of functional foods aimed at promoting health [296].

In summary, phytochemicals, such as polyphenols, omega-3-rich oils, fucoidans, agar, carrageenan, and quercetin, are increasingly recognized for their dual roles in improving both the quality and health benefits of food products. These compounds, sourced from plants and algae, contribute to enhanced shelf life, nutritional value, and functional properties while offering a wide range of health benefits, including antioxidant, antimicrobial, and cardiovascular protective effects. Some commercialized available examples of these ingredients include Ceamsea ™ from Ceamsa Ltd. [297], Fucoidan Extract from Swanson Ltd. [298], Agar-Agar from Algamar Ltd. [299], and Omega-3 formula from Kohbo Labs Ltd. [300].

## 6. Conclusions

This review uncovers the versatile functions of antioxidants in plant organisms and their involvement in protecting against OS. Several antioxidant compounds, such as flavonoids, carotenoids, and phenolic acids, promote plant resistance and are also beneficial for human health. A better understanding of these mechanisms may allow the optimization of agricultural practices and food supplies. Further research should be directed towards the more efficient use of plant antioxidants to improve their productive capacity and nutraceutical value. In this way, the integration of advanced omics technologies, such as metabolomics and transcriptomics, may improve the understanding of the biosynthetic pathways and regulatory networks of plant antioxidants. Furthermore, the convergence of plant biotechnology with sustainable agricultural practices offers promising avenues not only for increasing crop yields but also for enriching the nutritional and functional qualities of food products.

## Figures and Tables

**Table 2:** Chemopreventive activities of plant extracts and their phytochemicals: in silico, in vivo, and in vitro examples.

Plant Species	Extract	Phytochemicals	Activity	Analysis Method	Assay	Mechanism of Action	Results	Ref.
**Antioxidant activity**								
Garlic (*Allium sativum* L.)	ns	Allicin (purity > 90%)	Antitumor (cholangiocarcinoma)	CCK-8, FC, WB	In vitro/in vivo	STAT3 inhibition via SHP-1 upregulation	Suppressed proliferation, invasion, EMT, and tumor growth	[146]
ns	HS-1793	Resveratrol analogue	Antitumor (murine breast cancer)	LYM assays, DNA damage, Treg/TAM	In vivo	Inhibits immune suppression by Treg/TAM	Enhanced LYM proliferation, reduced Tregs, and decreased IL-10/TGF-*β*	[147]
Green tea *Curcuma longa* L.	ns	Catechins, curcumin	Antitumor (OSCC)	Histology, immunofluorescence, FC	In vivo	AP induction and anti-angiogenesis	Reduced tumor growth, increased apoptosis	[144]
*Melilotus officinalis* L.	ns	DC (coumarin derivative)	Anti-proliferative, gonad-safe	DC injection in BALB/c mice ovarian apoptosis, meiotic spindle	In vitro/In vivo	Cell cycle alteration without tubulin disruption	DC suppressed cell proliferation and increased AP in Vero and MCF-7 cells	[148,149]
*Polyalthia longifolia* L.	ME	Tetranorditerpene	Anticancer (prostate, leukemia cells)	Proteomic analysis	In vitro	ER stress activation, apoptosis	Inhibited tumor cell growth	[150]
*Fagonia cretica* L.	AqE	ns	Cytotoxic, induces cell cycle arrest	siRNA knockdown, MTT and FC, comet assay, WB	In vitro	Induction of DNA damage, activation of p53 and FOXO3a	Induced cell cycle arrest and AP in two phenotypic breast cancer cell lines	[151]
*Onobrychis argyrea* L.	ME (leaves)	Quinic acid, isoquercitrin, epicatechin, routine	Antioxidant, antidiabetic, anticancer	LC-MS/MS, DPPH, iron reduction, enzyme inhibition, XTT, FC	In vitro	ME induces apoptosis in HT-29 cells by disrupting mitochondrial membranes and activating caspases	Strong antioxidant and cytotoxic effect	[152]
*Elephantopus mollis* Kunth.	ME	3,4-di*-O-*caffeoyl quinic acid	Cytotoxicity, glucosidase inhibition	DPPH, FRAP, metal chelation, *β*-carotene, cytotoxicity	In vitro	Induces cell death in NCI-H23 cells by triggering apoptosis	High antioxidant capacity, induced apoptosis	[153]
*Thymus vulgaris* L.	MetE, EAE, ChE, BolE, AqE, PEE	Polyphenols, tannins, flavonoids, sterols/triterpenes	Antioxidant	DPPH, ABTS, ferrous ion chelation, CVT	In vitro	Radical scavenging, metal chelation	Strong antioxidant capacity correlated with phenol and flavonoid content	[154]
*Markhamia lutea* L.	Leaf extract	Flavonoids (*O-*glycosides)	Antioxidant, anti-AChE, /BChE, A*β*-42	DPPH, ORAC, iron reduction, FRAP	In silicoin vitro	Inhibits AChE, BuChE, and Aβ-amyloid-42	DPPH: 35.69 µg/mL, ORAC: 16,694.4 μM TE/mg, and iron chelation: 70.7 μM EDTA eq/mg	[155]
*Hertia cheirifolia* L.	Organic and EtOAc fraction	Total phenolics (100–250 mg GAE/g)	Antioxidant	DPPH, ABTS, FRAP, *β*-carotene	In vitro	Synergistic compound interaction	DPPH: 38.83 µg/mL, ABTS: 23.76 µg/mL, FRAP: 2628.87 µmol Fe^2+^ Eq/mL, and *β*-carotene: 58.91%	[156]
ns	AqE	QUE	Anticarcinogenic	WB, RT-PCR	In vitro	Downregulation of ROS, PKC, PI3K, and COX-2; upregulation of p53 and BAX	QUE modulated OS and apoptotic pathways in HepG2 cells	[157]
**Anti-inflammatory activity**								
*Tylophora ovata* L.	Natural and synthetic PAs	*O-*methyltylophorinidine (1, 1s)	Antitumor against TNBC	NFκB inhibition, 3D co-culture	In vitro	Stabilizes IκB*α*, blocks NFκB	Inhibited spheroid growth, surpassed paclitaxel	[158]
*Mangifera indica* L.	ns	Polyphenols	Anti-inflammatory, anticancer	Real-time PCR analysis and protein expression	In vitro	Modulates PI3K/AKT/mTOR, NF-κB, PARP-1, and Bcl-2	Reduced cancer cell growth by 90%	[159]
*Helicteres isora* L.	DCM-E/HeE	Rosmarinic acid	Anti-inflammatory, antioxidant	ELISAs	In vitro	Differentiation in cancer cells, shows no cytotoxic effect at high levels	Reduced TNF-*α*, PGE-2, and NO levels; highest COX-2 inhibition	[160]
*Waltheria indica* L.	Roots and aerial parts/CH_2_Cl_2_ extract	Flavonoids	Anti-inflammatory, chemopreventive	NF-κB inhibition, luciferase reporter assay, QR-inducing assay	In vitro	Induces phase 2 enzyme activity via QR induction assay	A total of 7/29 compounds showed inhibitory activity on the NF-κB pathway	[161]
*Commiphora leptophloeos* L.	Hydroalcoholic leaf extract	Phenolic acids and flavonoids	Anti-inflammatory	NO radical inhibition analysis, qPCR, physicochemical tests	In vitro/in vivo	Downregulates NF-κB and COX-2, reduces cytokines	Reduced inflammatory markers, promising for inflammatory bowel disease	[162]
*Matricaria chamomilla* L.	ns	*β*-Amyrin, *β*-eudesmol, *β*-sitosterol, apigenin, lupeol, quercetin, myricetin	Anti-inflammatory, anticancer	Proteome analysis, WB, qRT-PCR, thermophoresis	In silicoin vitro	Inhibition of NF-κB, reduces IL-1*β* and IL6 mRNA expression and G2/M cell cycle arrest	Cancer prevention, reduced pro-inflammatory cytokine expression	[163]
*Asparagus densiflorus meyeri* L	Root and aerial parts/DCM-E	Saponins, glycosides, sterols, triterpenes	Cytotoxic, anti-inflammatory	MTT assay, MCF-7 cell stimulation using TNF-*α*, RT-PRC	In vitro	Reduces NO release and NF-κB gene expression	Significant cytotoxicity (IC_50_ 26.13 μg/mL)	[164]
*Capparis cartilaginea* L.	Ethanolic leaf extract	Alkaloids, flavonoids, phenols, fatty acids, carotenes	Antioxidant, cytotoxic, anti-inflammatory	FBRC, FRAP, MTT assay, COX-1 inhibition	In vitro	Dose-dependent inhibition of thermally induced protein denaturation	Anti-inflammatory effects (IC_50_ 60.23 and 17.67 µg/mL) were better than standards	[165]
*Corchorus olitorius* L., *Amaranthus hybridus* L.	Hydroethanolic leaf extract	Tannins, flavonoids, phenolics, terpenoids, cardiac glycosides, coumarins	Pro-estrogenic, anti-inflammatory	Phytochemical and ELISA analyses	In vivo	Lowers IL-6 and inhibits proliferation by binding phytoestrogens to ER-*β*	Antioxidant because of its high tannin content/reduction in tumor size and incidence	[166]
*Euphorbia hirta* L.	Whole extract	Phytol, fatty acids, 5-HMF	Anti-inflammatory	NO production	In vitro	Suppression of PG generation	Inhibition of iNOS directly involved in inflammation	[167]
**Antidiabetic activity**								
*Tradescantia pallida* L.	Leaf extract	Syringic acid, p-coumaric acid, morin, catechin	Glycosylation and hemoglobin activity	α-Amylase assay	In vitro	Glycosylation inhibition non-enzymatically	Boosted insulin production, revitalized *β*-cells, inhibited AGEs, stimulated glucose transporters and AMPK	[168]
*Cissampelos capensis* L.	Leaf, stem, and rhizome	Glaziovine, pronuciferine, cissamanine	Antihyperglycemic	α-Amylase assay	In vitro	Enzyme inhibition pathway	Reduced glucose levels	[169]
*Phyllanthus emblica* L.	ns	Flavonoids	Antihyperglycemic	Molecular docking assay	In silico	Hypoglycemic action, reduces relative risk of T2D, PPAR inhibition of T2D	High binding affinity and selectivity for T2D therapeutic targets	[170]
*Ocinum sanctum* L.	Leaves	Eugenol	Antihyperglycemic	ELISA, RIA, and Neutral Red assay	In vitro	Physiological pathway	Decreased plasma glucose levels in T2D, increased islet insulin secretion, perfused pancreas	[171]
*Ocinum basilicum* L.	Leaves	TPC and FC	Antihyperglycemic	Enzyme inhibitory activity assay	In vitro	Enzyme inhibition pathway (*α*-glucosidase, *α*-amylase, DPP-IV, PTP1B, and SGLT2)	Inhibition of intestinal sucrase, maltase, and porcine pancreatic *α-amylase*	[172]
*Derris elliptica* L.	Leaves	QUE and ceramide	Antihyperglycemic	Biochemical analysis and histopathology study	In vivo	Enzyme inhibition pathway	Increased insulin secretion, protected pancreatic *β*-cells from oxidative stress	[173]
*Carica papaya* L.	Seeds	Hexadecanoic acid methyl ester, 11-ODA oleic acid	Antihyperglycemic	α-Amylase and α-glucosidase inhibition assay	In vitro	Enzyme inhibition pathway	Reduced glucose levels	[174]
*Rhazya stricta* L.	Roots	Hexadecanoic acid, methyl ester	Antihyperlipidemic, hepatoprotective	DPP-IV, α-amylase, α-secretase inhibition assay, GLP-1 measurement	In vitro/in vivo	Enzyme inhibition pathway	Reduced blood glucose and HbA1c, reduced cholesterol and triglyceride levels, reduced liver enzyme activity	[175]
*Halooxylon stocksii* L.	Root and aerial parts	8-ODA methyl ester	Antidiabetic	α-Amylase and *α*-glucosidase assay	In vitro	Enzyme inhibition pathway	Reduced glucose levels	[176]
**Anti-obesity activity**								
*Rosa centifolia* L.	Petals	Ellagic acid (polyphenols)	Lipid metabolism improvement	PCR	In vivo	Suppression of lipid synthesis, inhibition of intestinal absorption, downregulation of *Scd1* and *Hmcgr* mRNAs in the liver	Reduced body weight and adipose tissue, increased fecal triglycerides, lipid, and cholesterol metabolism	[177]
*Rheum rhabarbarum* L.	ns	Emodin, rhein (anthraquinones)	Lipid-lowering	ELISA and histological evaluation	In vitro/in vivo	FAS-ACC production prevention through decreased PPARγ and C/EBP*α* expression, reduced lipid accumulation	Body weight and adipose tissue reduction	[178]
*Brassica juncea* L.	ns	Sinigrin (glucosinolate)	Anti-obesity	Cell culture and XTT assay, WB, histological analysis	In vitro/in vivo	Reduces expression of adipogenic and lipid synthesis proteins	Inhibited lipid accumulation in 3T3-L1 and decreased eWAT mass in obese mice fed a high-fat diet	[179]
*Anthophycus longifolius* L.	ns	Rhodomycinone, salsolinol, 5-HCO, 2-COS, demethylalangiside	Anti-obesity, anti-hyperglycemia	α-Amylase, *α*-glucosidase, pancreatic lipase assay	In vitro	Enzyme inhibition pathway	Delayed lipid, CH digestion, and absorption	[180]
*Solanum xanthocarpum* L.	Fresh and dry leaves	Solasodine, carpesterol, *β*-sitosterol, diosgenin	Hypoglycemic, hepatoprotective, hypotensive	Pancreatic lipase inhibition assay, MTT	In vitro	ns	At 62.5 µg/mL, the fresh leaf extract reduced cancer cell viability by 50%	[181]
*Rumex rothschildianus* L.	Acetone fraction	Flavonoids, phenolics	Anti-*α*-amylase, anti-*α*-glucosidase, anti-lipase	Lipase inhibition activity	In vitro	Inhibits OS, *α*-amylase, *α*-glucosidase, and lipase	Strong lipase inhibition (acetone fraction IC_50_ 26.3 μg/mL), close to orlistat (IC_50_ 12.3 μg/mL)	[182]
**Neuroprotective activity**								
*Paeonia ostii*	Stamen	(+)-3′′-methoxy-oxylactiflorin	Anti-inflammatory	Molecular docking, NO inhibition assay	In vitro/in silico	Inhibition of NO production by binding with protein COX-2	Reduced NO production to values of EC_50_ 3.02 μM	[183]
*Phyllanthus emblica*	Fruit extract	ns	Anti-inflammatory	NO inhibition assay	In vivo	Reduces IL-1β and TNF-α/increases expression of 5-HT1D, 5-HT2A, and D2 receptors	Amelioration of social interaction, social affiliation, anxiety, and motor coordination	[184]
*Tabebuia impetiginosa*	Leaves	Iridoids and organic acids	Anti-inflammatory	AChE inhibitory activity, LA detection, Y-maze test, PA assay	In vitro/in vivo	CP attenuates cognitive impairment, effects rat performance in Y-maze and PA tests	Reduced CP-induced chemo-brain, restored hippocampal function	[185]

Abbreviations: CCK-8: Cell Counting Kit-8; STAT3: Signal Transducer and Activator of Transcription 3; WB: Western Blotting; FC: Flow Cytometry; SHP-1: Src Homology 2 Domain-Containing Phosphatase-1; EMT: Epithelial–Mesenchymal Transition; Treg: Regulatory T Cell; TAM: Tumor-Associated Macrophage; LYM: Lymphocyte; IL-10: Interleukin-10; TGF-β: Transforming Growth Factor-Beta; ME: Methanol Extract; OSCC: Oral Squamous Cell Carcinoma; AP: apoptosis; DC: Dicoumarol; BALB/c: Bagg Albino Laboratory Strain/c; Vero: African Green Monkey Kidney Epithelial Cells; MCF-7: Human Breast Cancer Cells; siRNA: Small Interfering RNA; MTT assay: 3-(4,5-Dimethylthiazol-2-yl)-2,5-Diphenyltetrazolium Bromide Assay; p53: tumor protein p53; FOXO3a: Forkhead Box O3; QUE: quercetin; ROS: reactive oxygen species; OS: oxidative stress; PKC: Protein Kinase C; PI3K: Phosphatidylinositol 3-Kinase; COX-2: Cyclooxygenase-2; RT-PCR: Reverse Transcription Polymerase Chain Reaction; HepG2 cells: Human Liver Cancer Cell Line; AMPK: Adenosine Monophosphate-Activated Protein Kinase; AGES: advanced glycation end products; T2D: Type 2 Diabetes; PPAR: Peroxisome Proliferator-Activated Receptor; NFκB: Nuclear Factor Kappa B; IκBα: Inhibitor of Nuclear Factor Kappa B Alpha; HT-29 cells: Human Colorectal Adenocarcinoma Cell Line; DPPH: 2,2-Diphenyl-1-picrylhydrazyl; FRAP: Ferric-Reducing Antioxidant Power; NCI-H23 cells: Human Lung Adenocarcinoma Cell Line; ORAC: Oxygen Radical Absorbance Capacity; ER: Endoplasmic Reticulum; HeE: Hexane Extract; DCM-E: Dichloromethane Extract; MetE: Methanolic Extract; EAE: Ethyl Acetate Extract; ChE: Chloroform Extract; BolE: Butanol Extract; AqE: aqueous extract; PEE: Petroleum Ether Extract; CVT: Cyclic Voltammetry Technique; PI3K/AKT/mTOR: Phosphoinositide 3-Kinase/Protein Kinase B/Mechanistic Target of Rapamycin; PARP-1: Poly (ADP-ribose) Polymerase 1; Bcl-2: B-cell Lymphoma 2; TNF-α: tumor necrosis factor-alpha; THP-1 cells: Human Monocyte Cell Line; ELISA: Enzyme-Linked Immunosorbent Assay; QR: Quinone Reductase; NO: nitric oxide; 5-HMF: Hydroxymethyl-2-furancarboxaldehyde; iNOS: Inducible Nitric Oxide Synthase; PG: prostaglandin; RIA: Radioimmune Assay; TPCs: Total Polyphenol Compounds; FC: flavonoid content; 11-ODA: 11-Octadecanoic Acid; HbA1c: Glycosylated Hemoglobin; PCR: Polymerase Chain Reaction; FAS: Fatty Acid Synthase; ACC: Acetyl-CoA Carboxylase; 5-HCA: 5-Hydroxyconiferyl; 2-COS: 2-Caffeoylisocitrate; DPP-IV: Dipeptidyl Peptidase-IV; PTP1B: Protein Tyrosine Phosphatase 1B; SGLT2: Sodium–Glucose Co-Transporter 2; qPCR: Quantitative Polymerase Chain Reaction; FBRC: Ferric-Bipyridine-Reducing Capacity of Total Antioxidant; ER-β: Estrogen Receptor Beta; eWAT: epididymal white adipose tissue; IL-1β: Interleukin 1 Beta; TNF-α: tumor necrosis factor-alpha; 5-HT1D: Serotonin 1D Receptor; 5-HT2A: Serotonin 2A Receptor; D2: Dopamine 2 Receptor; AChE: Acetylcholinesterase; LA: Locomotor Activity; PA: Passive Avoidance; ns: Not Specified.

**Table 3:** Nutraceutical applications and functionality.

Nutraceutical	Source	Applications	Functionality	Health Benefits	Ref.
Pectin	Fruits (apple, citrus…)	Jams, jellies, dairy products	Gelling agent, thickener	Anticancer, immunomodulatory, anti-inflammatory, cholesterol-lowering	[260]
Inulin	Chicory root	Low-fat foods, fiber supplements	Prebiotic, fat replacer	Gut microbiota regulation, lipid metabolism regulation, mineral absorption enhancement, anti-inflammatory	[261]
Cellulose	Plants	Low-fat foods, plant-based meats, bakery products	Stabilizer, thickener	Gut microbiota regulation, cholesterol reduction, blood glucose level regulation, anti-inflammatory	[239]
Wheat bran	Wheat	Cereals, bread, bakery products	Texture enhancer, fiber source	Gut microbiota regulation, cancer risk reduction, cardioprotective	[262]
Psyllium husk	*Plantago ovata* seeds	Fiber supplement, cereals	Fiber source, thickener	Antidiabetic, reduces cholesterol levels, aids in gastrointestinal health	[263,264]
Catechins	Green tea leaves	Beverages, supplements, snacks	Antioxidant, antimicrobial	Antioxidant, anti-inflammatory, antiviral, anti-obesity	[265,266]
Grape seed extract	Grape seeds	Beverages, supplements	Antioxidant, antimicrobial	Anti-inflammatory, antioxidant, cardioprotective, antimicrobial, anticancer	[267]
β-carotene	Carrots, sweet potatoes	Supplements, snacks, beverages, candies	Colorant	Antioxidant, supports immune function	[268]
Anthocyanins	Berries, red cabbage	Supplements, snacks, beverages, candies	Colorant	Antioxidant, anti-inflammatory, antidiabetic, anti-obesity	[269]
Betanins	Beetroot	Supplements, snacks, beverages, candies	Colorant	Antioxidative, anti-inflammatory, antidiabetic, potential anticancer benefits	[248]
Curcumin	Turmeric root	Supplements, snacks, beverages, candies	Colorant	Antioxidant, anti-inflammatory, anticancer, and immune-regulatory properties	[270]
Resveratrol	Grapes	Beverages, supplements	Antioxidant, antimicrobial	Antioxidant, anti-inflammatory, anticancer, cardioprotective	[271]
Fucoidans	Brown algae	Supplements, fortified foods	Gelling agent, thickener	Antioxidant, anti-inflammatory, anticoagulant, antitumor, antiviral	[272]
Agar	Red algae	Jellies, jams, candy, plant-based gelatin	Gelling agent, texture enhancer	Antioxidant, antiviral, antibacterial, prebiotic, antitumor	[273]
Carrageenan	Red algae	Jellies, jams, candy, plant-based gelatin	Thickener, gelling agent	Cardioprotective, anticancer, antiviral, anticoagulant, antioxidant	[274]
Quercetin	Onions, apple peels	Supplements, fortified foods	Antioxidant, preservative	Anti-inflammatory, antimicrobial, anticancer, cardioprotective	[275,276]

## Data Availability

No new data were created or analyzed in this study. Data sharing is not applicable to this article.
